# Weighted Genomic Best Linear Unbiased Prediction for Carcass Traits in Hanwoo Cattle

**DOI:** 10.3390/genes10121019

**Published:** 2019-12-06

**Authors:** Bryan Irvine Lopez, Seung-Hwan Lee, Jong-Eun Park, Dong-Hyun Shin, Jae-Don Oh, Sara de las Heras-Saldana, Julius van der Werf, Han-Ha Chai, Woncheoul Park, Dajeong Lim

**Affiliations:** 1 Division of Animal Genomics and Bioinformatics, National Institute of Animal Science, Rural Development Administration, Wanju 55365, Korea; irvinelopez@korea.kr (B.I.L.); jepark0105@korea.kr (J.-E.P.); hanha@korea.kr (H.-H.C.); wcpark1982@korea.kr (W.P.); 2 Department of Animal Science and Biotechnology, Chungnam National University, Daejeon 34134, Korea; slee46@cnu.ac.kr; 3 Department of Animal Biotechnology, Chonbuk National University, Jeonju 54896, Korea; sdh1214@gmail.com (D.-H.S.); oh5ow@naver.com (J.-D.O.); 4 School of Environmental and Rural Science, University of New England, Armidale 2351, Australiajvanderw@une.edu.au (J.v.d.W.)

**Keywords:** genomic prediction, weighted GBLUP, carcass traits, Hanwoo cattle

## Abstract

The genomic best linear unbiased prediction (GBLUP) method has been widely used in routine genomic evaluation as it assumes a common variance for all single nucleotide polymorphism (SNP). However, this is unlikely in the case of traits influenced by major SNP. Hence, the present study aimed to improve the accuracy of GBLUP by using the weighted GBLUP (WGBLUP), which gives more weight to important markers for various carcass traits of Hanwoo cattle, such as backfat thickness (BFT), carcass weight (CWT), eye muscle area (EMA), and marbling score (MS). Linear and different nonlinearA SNP weighting procedures under WGBLUP were evaluated and compared with unweighted GBLUP and traditional pedigree-based methods (PBLUP). WGBLUP methods were assessed over ten iterations. Phenotypic data from 10,215 animals from different commercial herds that were slaughtered at approximately 30-month-old of age were used. All these animals were genotyped using customized Hanwoo 50K SNP chip and were divided into a training and a validation population by birth date on 1 November 2015. Genomic prediction accuracies obtained in the nonlinearA weighting methods were higher than those of the linear weighting for all traits. Moreover, unlike with linear methods, no sudden drops in the accuracy were noted after the peak was reached in nonlinearA methods. The average accuracies using PBLUP were 0.37, 0.49, 0.40, and 0.37, and 0.62, 0.74, 0.67, and 0.65 using GBLUP for BFT, CWT, EMA, and MS, respectively. Moreover, these accuracies of genomic prediction were further increased to 4.84% and 2.70% for BFT and CWT, respectively by using the nonlinearA method under the WGBLUP model. For EMA and MS, WGBLUP was as accurate as GBLUP. Our results indicate that the WGBLUP using a nonlinearA weighting method provides improved predictions for CWT and BFT, suggesting that the ability of WGBLUP over the other models by weighting selected SNPs appears to be trait-dependent.

## 1. Introduction

The Hanwoo (Korean native cattle) is well known for extensive marbling, tenderness, juiciness, and the characteristic flavor of its beef [1]. Progress in its production has been favorable due to the constant genetic improvement of animals. At present, the Hanwoo breeding program considers the estimated breeding values for carcass traits, such as backfat thickness (BFT), carcass weight (CWT), eye muscle area (EMA), and marbling score (MS), as major selection criteria in choosing the parents for the next generation. With the steadfast advances in high-throughput genotyping and the constant availability of molecular data, commercial application of genomic selection has been rapidly adopted for the improvement of livestock species [2,3]. Nonetheless, this genomic selection has not yet been fully implemented in Hanwoo breeding schemes, though it is thought to be advantageous in small populations [4,5].

The genomic best linear unbiased prediction (GBLUP) method has been widely used in routine genomic evaluation because it is easier to implement and less computationally demanding than the Bayesian Alphabet methods. However, GBLUP usually assumes that all single nucleotide polymorphisms (SNPs) explain the same fraction of genetic variance [6,7], which is unlikely in the case of traits with different architectures and those influenced by major SNP. To overcome these limitations of GBLUP, Zhang et al. (2010) [8] initially proposed the addition of unequal weights for all SNPs in a method called weighted GBLUP (WGBLUP) and reported higher accuracies than the unweighted counterpart. Many others explored the use of various weights for GBLUP in a single-trait evaluation, either weighting SNPs individually or assigning a common weight to adjacent SNPs [9,10]. Zhang et al. (2016) [10] used an iterative weighting procedure with simulation data and observed that the use of a common weight for a window of 20 SNPs that sums or averages the SNP variance improved the accuracy of prediction. Single-step WGBLUP performance has been reported to be better than the unweighted counterpart for protein content [11], milk production traits, udder type traits, and somatic cell scores [12] in French dairy goats.

The objective of this study was to investigate linear and different nonlinearA SNP weighting methods under the WGBLUP models with the hope of increasing the accuracy of genomic breeding value (GEBV) prediction for carcass traits in the commercial Hanwoo breeding population. The accuracies of the predicted breeding values derived from the aforesaid method were then compared with GBLUP and the traditional pedigree-based method (PBLUP).

## 2. Materials and Methods

### 2.1. Phenotypic and Genotypic Data

The phenotypic and genotypic data employed in this study were derived from 10,215 Hanwoo cattle born between April 2006 and March 2016 from different commercial herds located across South Korea. The animal population was composed mostly of steers (9856), castrated at about 4 months of age. Animals were reared in sheds containing multiple pens (8–10 head/pen), were fed a concentrate mixture and a rice straw-based ration, and were slaughtered at approximately 30 months of age. All these animals were cared for according to the appropriate animal health and welfare guidelines approved by the Animal Care and Use Committee of the National Institute of Animal Science, Rural Development Administration, Korea. Ethics approval for this study was given by the Animal Care and Use Committee of the National Institute of Animal Sciences under the code 2018-293 (from 21 May 2018). The total number of animals in the pedigree was 29,176, from seven (7) generations with 428 bulls and 13,925 dams. Thirty-six (36) animals were found to be inbred, with an average inbreeding coefficient of 0.04. Pedigree structures and inbreeding coefficients were obtained using WOMBAT software [13].

The traits analyzed in this work were backfat thickness (BFT), carcass weight (CWT), eye muscle area (EMA), and marbling score (MS). BFT, EMA, and MS were measured in the rib eye muscle at the 12th and 13th rib junction, while cold CWT was weighed after chilling for about 24 h. Marbling score was graded using a 9-point scale following the Korean Beef Marbling Standard [14]. The statistics for the carcass traits are summarized in Table 1.

Meat tissue samples collected from the same muscle were genotyped using the customized Hanwoo 50K SNP Chip (Illumina, South Korea). The SNPs with call rates less than 0.9, minor allele frequencies less than 0.01, and deviations from the Hardy–Weinberg equilibrium with a *p* < 10^−6^ were excluded from the genotype data set. Parent-progeny pairs were tested for discrepant homozygous SNP, and the progeny was removed when the conflict rate reaches greater than 1%. After quality control, 37,549 SNPs remained for further analyses.

### 2.2. Statistical Models

Here, the traditional GBLUP method with a pedigree-based relationship matrix, the GBLUP method, and WGBLUP methods with a genomic relationship matrix constructed from marker information were used to predict breeding values. GBLUP and WGBLUP assume equal and unequal weights for all SNPs, respectively. The animals were split into training and validation datasets according to year of birth, and corrected phenotypes in the validation dataset were used to compute the accuracy of predictions.

#### 2.2.1. Pedigree Best Linear Unbiased Prediction (PBLUP)

For traditional pedigree-based genetic evaluations, the following single-trait animal model was fit:

(1) y=Xb+Za+e,. 

where **y** is a vector of observations; **b** is the vector of fixed effects of herds (162 herds), year-month of birth (82 levels), slaughter year (9 levels), slaughter place (52 levels), and the sex and age as a covariate; **a** is the vector of additive genetic effects following a normal distribution $N\left(0,\;A\sigma_{a}^{2}\right)$. with $\sigma_{a}^{2}$. being the additive genetic variance and **A** being the pedigree-based relationship matrix of individuals. **X** and **Z** are indices matrices associating **b** and **a** with **y**, respectively. **e** is the vector of the random residual effect that follows a normal distribution $N\left(0,\;I\sigma_{e}^{2}\right)$ with $\sigma_{e}^{2}$. being residual variance and **I** an identity matrix. A multi-trait animal model was used to estimate the covariance components for calculating genetic correlations between traits.

#### 2.2.2. Genomic Best Linear Unbiased Prediction (GBLUP)

Here, we used corrected phenotypes (Yc) as response variables, which were computed using the pedigree-based model presented above (Yc = **y** − **Xb**). The GBLUP model uses a genomic relationship matrix (**G**) created from the SNP markers instead of the pedigree-based relationship (**A**). Following VanRaden (2008) [6], the **G** matrix was created as

(2) $$G=\frac{MM^{\prime }}{\sum_{i=1}^{m}2p_{i}{\left(1-p_{i}\right)}_{\;}^{\;}},$$

where **M** is a matrix of centered genotypes; m is the number of SNPs, and $p_{i}$. is the frequency of an allele *i*-th SNP.

#### 2.2.3. Weighted Genomic Best Linear Unbiased Prediction (WGBLUP)

In the weighted GBLUP, model and inferences were identical as the GBLUP with the matrix **G** was constructed differently. The creation of the **G** matrix shown above assumes that each SNP explains the same amount of genetic variance. In the WGBLUP approach developed by Wang et al. (2012) [15], major SNPs with relatively large effects were included as weighted **G** (G*). This genomic relationship matrix G* is constructed as follows:

(3) $$G^{*}=\;\frac{MDM^{\prime }}{\sum_{i=1}^{m}2p_{i}{\left(1-p_{i}\right)}_{\;}^{\;}}$$

where **M**, *p_i_*, and *m* are the same as **G** and **D** in a diagonal matrix, where each element of the diagonal corresponds to SNP weights. Wang et al. (2012) [15] derived conversions of GEBV under the single-step GBLUP model into SNP effects as

(4) $$\hat{u}=DZ^{\prime }\;G^{-1}\hat{g,}$$

where $\hat{u}$. is a vector of estimated SNP effects and $\hat{g}$. is a vector of GEBV from genotyped animals only. The weight for SNP *i* was calculated as $u_{i}^{2}$. in the training group.

##### Weighted GBLUP with Linear Weights

The iterative steps in the algorithm of the WGBLUP approach following Wang et al. (2012) [15] are as follows:

1. Set parameters to t = 1, $D_{\left(t\right)}=I$, $G_{\left(t\right)}=D_{\left(t\right)}Z^{\prime }\lambda$, where $\lambda =\frac{1}{\sum_{i=1}^{m}2p_{i}{\left(1-p_{i}\right)}_{\;}^{\;}}$;
2. Calculate GEBVusing the GBLUP approach;
3. Compute SNP effects as  u ^(t)= λD(t)Z´G(t)−1 a ^g;
4. Calculate SNP weight as $d_{i\left(t+1\right)}=u_{i}^{2},$ where *i* is the *i*-th SNP.
5. Normalize SNP weights to keep the total genetic variance constant:
  $$D_{\left(t+1\right)}=\;\frac{\mathrm{tr}(D_{\left(1\right)})}{\mathrm{tr}(D_{\left(t+1\right)})}D_{\left(t+1\right)}$$
6. $G_{\left(t+1\right)}={\mathrm{ZD}}_{\left(t+1\right)}Z´\lambda$ was calculated;
7. t = t + 1 and loop to step 2.

Here, GEBV was replaced by direct genomic values (DGV) in step 3. According to Lourenco et al. (2015) [16], DGV is a more relevant starting point because genotyped populations may be comprised of animals with different levels of accuracy.

##### Weighted GBLUP with NonlinearA Weights

In step 4, SNP weights were calculated as $d_{i\left(t+1\right)}={\;\mathrm{CT}}^{\frac{\left|{\hat{u}}_{i}\right|}{sd\left(\hat{u}\right)}-2}$, adapted from VanRaden (2008) [6], where CT represents the departure from normality and *sd* is the standard deviation. A limit was used on the exponent of CT to avoid extreme values. VanRaden (2008) [6] used 1.25 for the departure from normality. In our study, different values for CT were tested (1.125 and 1.25) with different exponential limits of 5, 10, and 20.

The abovementioned procedures were run for ten iterations. The convergence criterion was set to 1 × 10^−15^. All analyses were conducted using the BLUPF90 software family [17]. Following the estimation of variance components using AIREMLF90, phenotypes were corrected using PREDICTF90, genomic quality was controlled using PREGSF90, GEBV was predicted using BLUPF90, and SNP effects for WGBLUP were calculated using the postGSf90 software [17].

### 2.3. Validation

The predictive abilities of all the methods were evaluated by splitting the records into training and validation dataset by the date of 1 November 2015. The validation set included 1652 animals born after the cut-off date, while the training set was composed of 8563 animals. Phenotypes of animals under the validation set were assumed to be unknown. The accuracy of genomic prediction was evaluated by the correlation of the EBV or GEBV from the validation and the corresponding phenotypes corrected for fixed effects divided by the square root of the heritability of the trait.

(5) $$\mathrm{acc}=\frac{\mathrm{cor}\left[\left(G\right)\mathrm{EBV},\;\mathrm{Yc}\right]}{\sqrt{h^{2}}}$$

In addition, the bias of genomic prediction was assessed using the regression of corrected phenotypes on GEBV. Regression coefficients close to one are considered the most desirable.

## 3. Results and Discussion

### 3.1. Estimates of Genetic Parameters

The heritability estimates for carcass traits in Hanwoo cattle were medium to high, as presented in Table 1. Heritability estimates for BFT, CWT, EMA, and MS were 0.36(0.02), 0.37(0.02), 0.35(0.02), and 0.45(0.03), respectively. These results were in the range of previously reported estimates. Lee et al. (2018) [18] reported heritability estimates of 0.31(0.02), 0.42(0.02), 0.34(0.02), and 0.43(0.02) for BFT, CWT, EMA, and MS, respectively. Moreover, Hwang et al. (2014) [19] reported slightly higher heritability estimates of 0.39(0.07), 0.50(0.07), 0.40(0.07), and 0.56(0.07) for BFT, CWT, EMA, and MS, accordingly. The genetic and phenotypic correlations between traits are shown in the Supplementary File, Table S1. Estimates of genetic correlations between CWT and BFT (0.14 ± 0.10), CWT and MS (0.30 ± 0.09), and BFT and MS (0.12 ± 0.10) were low yet positive. On the other hand, EMA was found to have a moderate positive correlation with CWT (0.50 ± 0.09) and MS (0.56 ± 0.08), while it showed a low negative correlation with BFT (−0.19 ± 0.10). These estimates of genetic correlation among carcass traits were in agreement with those reported in previous works [20,21].

### 3.2. Predictive Accuracy and Unbiasedness with WGBLUP Methods over Iterations

**Figure 1:**
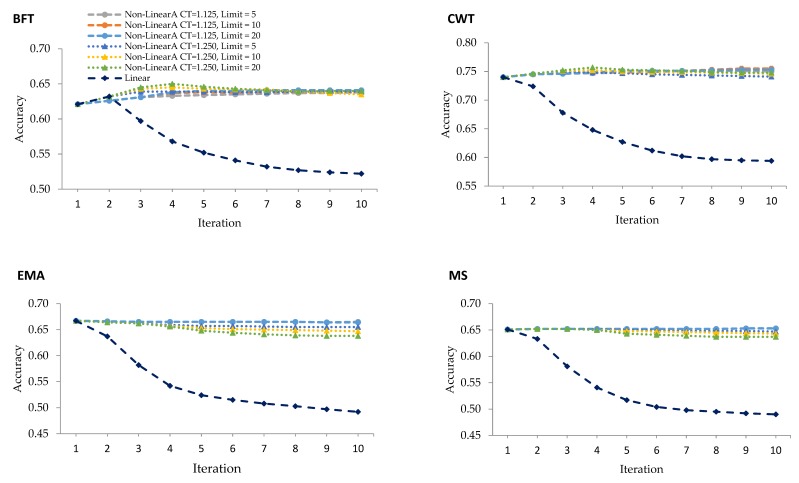
Predictive accuracies for validation animals using linear weighting and various nonlinearA weighting methods with different CT (departure from normality) values and exponent limits for backfat thickness (BFT), carcass weight (CWT), eye muscle area (EMA), and marbling score (MS).

Being an iterative weighting method, WGBLUP was performed in ten iterations where the SNP weights were equal to 1 in the first iteration corresponding to that of the GBLUP. Figure 1 demonstrates the accuracy of genomic prediction using linear and different nonlinearA weighting methods over ten iterations. The WGBLUP using the nonlinearA weighting method shows an enhanced accuracy of prediction for BFT and CWT, while no improvement for EMA and MS was noted over the iterations. The genomic prediction accuracies obtained in the nonlinearA weighting methods are higher than those of the linear weighting for all traits. Moreover, unlike with the linear method, no sudden drop in the accuracy was noted after the peak was reached in nonlinearA methods. 

Even after iteratively recomputing the SNP effects and the GEBV, there were still no improvements in the accuracy using the linear weighting method for all carcass traits, except for BFT. The highest accuracy using the linear weighting method was at the first iteration for CWT, EMA, MS, and the second iteration for BFT. However, the accuracy for BFT deteriorated over the succeeding iterations. Zhang et al. (2016) [10] purported that the decline in accuracy with iteration was the result of the continuous addition of weights to the SNP with large effects while shrinking the SNP with small influence, which, in turn, caused the gradual decrease in the accuracy of GEBV with every iteration. In a study using simulation, Wang et al. (2012) [15] reported the highest accuracy at the second iteration, which gradually decreased and remained constant after more iterations using a linear weighting method under the ssGBLUP model. These discrepancies between their work and our results could be due to the fact that the data we analyzed concern traits with different genetic architectures.

The nonlinearA weighting method proposed by VanRaden (2008) [6] was used in this study and two CT values with different exponential limits were evaluated. With nonlinearA methods, the inflection point came earlier with a CT value of 1.25 and exponent limit of 20, as observed in BFT and CWT (Table 2). Moreover, the accuracies remained stable after the peaks were reached at the fourth iteration with the said CT value and exponent limit for BFT and CWT. This method prevents the drastic decrease in accuracy by limiting the maximum SNP weights. In the 100 quantitative trait loci (QTL) scenario carried out in the work of Zhang et al. (2016) [10], the nonlinearA weighting method with CT values of 1.25 increased at iteration 3 and remained constant for the succeeding iterations. 

**Figure 2:**
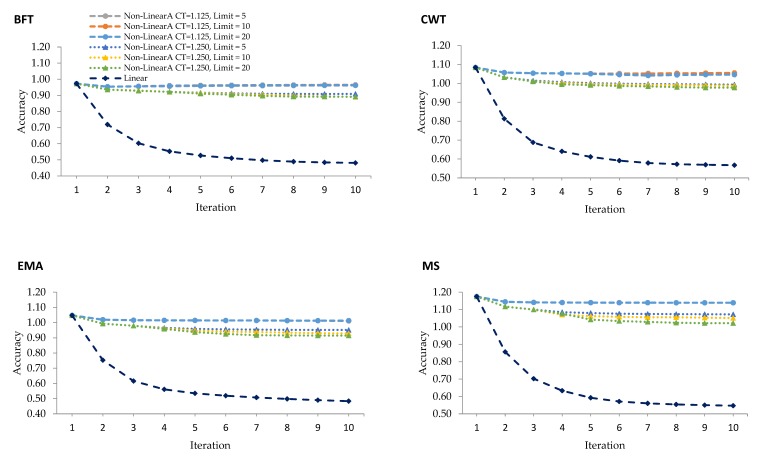
Regression coefficients of corrected phenotypes on GEBV from WGBLUP using linear and various nonlinearA weighting methods with different CT values and exponent limits for backfat thickness (BFT), carcass weight (CWT), eye muscle area (EMA), and marbling score (MS).

Figure 2 establishes the unbiasedness of genomic prediction using different weighting in the calculation of SNP weights in WGBLUP over ten iterations. Linear weighting methods tend to give upward biases over iterations. This was particularly true with the rapid decline of unbiasedness between iterations 1 and 2 in all traits under study. This was in contrast to the unbiasedness observed when using the nonlinearA weighting method that gradually decreased after iteration 1 and remained constant over iterations. As this particular method assigns more weight to SNP with effects but not to those with no effects, it tends to introduce biases into the GEBV [10].

**Figure 3:**
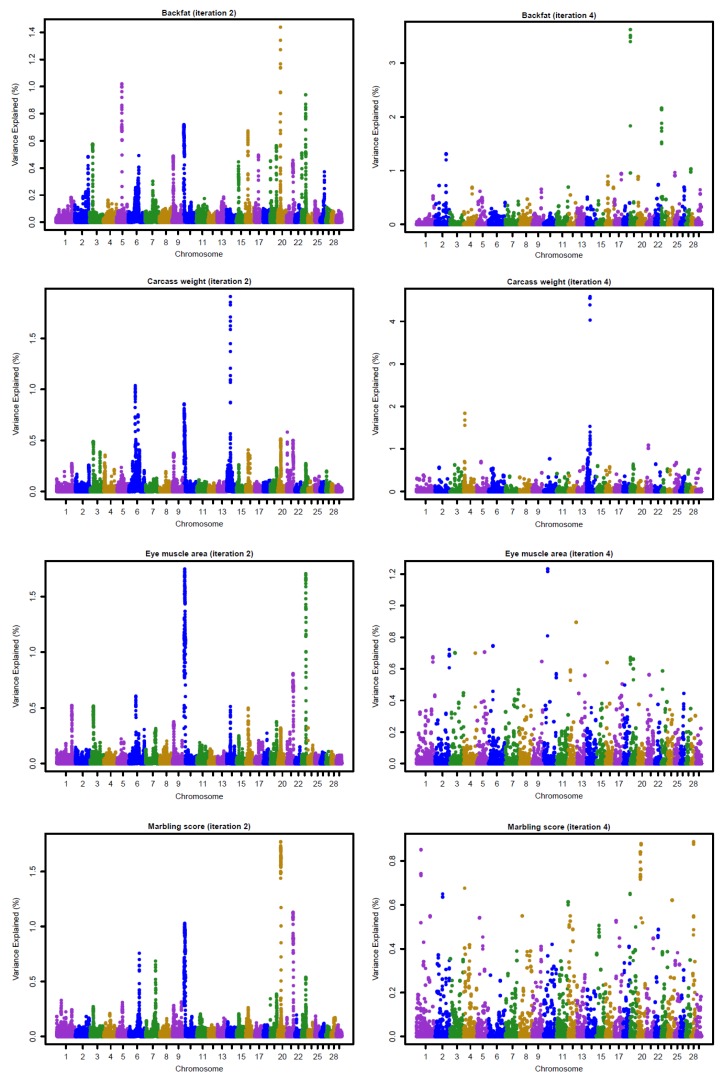
Proportion of variance (%) explained by SNP for carcass traits at iterations 2 and 4 using a linear weighting method with the weighted GBLUP approach.

**Figure 4:**
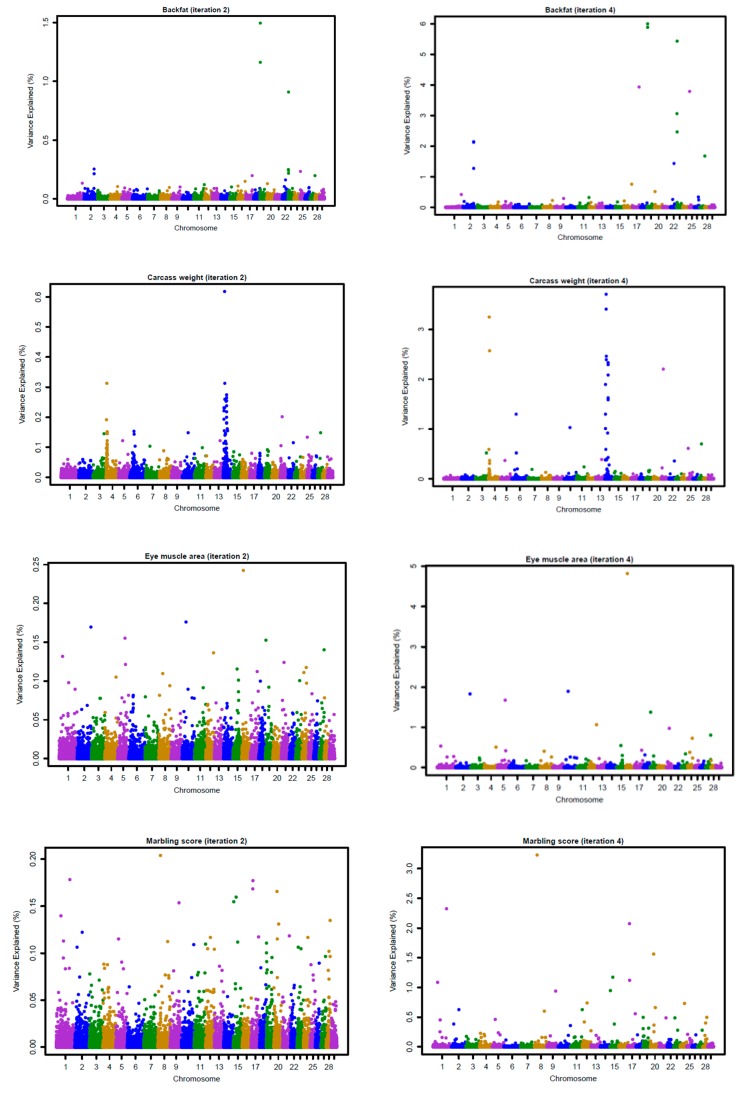
Proportion of variance (%) explained by SNP for carcass traits at iterations 2 and 4 using a nonlinearA (CT = 1.25, limit = 20) weighting method with the weighted GBLUP approach.

Figure 3 and Figure 4 show the Manhattan plots for SNP effects at iterations 2 and 4 for four carcass traits of Hanwoo cattle using the linear and nonlinearA weighting methods, respectively. The results confirm that BFT and CWT are influenced by major SNPs. The shrinkage of SNP effects using the nonlinearA weighting method was also found to be greater than that of the linear method. Moreover, the results reveal that CWT is controlled by few QTLs with large effects, especially on chromosome 4, 6, and 14 (Figure 1, iteration 4). For BF, high SNP effects were attributed to some SNP located on chromosome 19 and 23 (Figure 1, iteration 4).

### 3.3. Predictive Accuracy with PBLUP, GBLUP, and WGBLUP Models

The accuracies and biases of genomic prediction for carcass traits using PBLUP, GBLUP, and WGBLUP models are presented in Table 2. The accuracies derived from the WGBLUP method using linear weighting at the second iteration and nonlinearA weighting with a CT value of 1.25 and an exponential limit of 20 at the fourth iteration for BFT and CWT, and the second iteration for EMA and MS, were compared with those of PBLUP and GBLUP. It was noted that the accuracies of prediction for GBLUP were significantly higher than those of the PBLUP method. The average accuracies using PBLUP were 0.37, 0.49, 0.40, and 0.37, and 0.62, 0.74, 0.67, and 0.65 using GBLUP for BFT, CWT, EMA, and MS, respectively. The accuracies reported in this work were higher than those previously registered for Hanwoo cattle [4,5], which can be attributed to the higher number of genotyped animals in the reference population. Furthermore, these accuracies of genomic prediction were further increased to 4.84% and 2.70% for BFT and CWT, respectively by using the nonlinearA method under the WGBLUP model. For EMA and MS, WGBLUP results were as accurate as those of the GBLUP. These results suggest that the ability of WGBLUP over the other models by weighting selected SNPs appears to be trait-dependent.

The genomic selection in Hanwoo beef cattle has already been evaluated in small populations, although it has been not fully implemented in practical breeding. After a comparison of the accuracies of genomic prediction using GBLUP, BayesC, and BayesL in 1183 genotyped Hanwoo cattle, Mehrban et al. (2017) [4] reported similar accuracies for BFT, EMA, and MS for the three methods, with a 7% increase in the CWT using the BayesC. Moreover, Lee et al. (2017) [5], who also assessed genomic prediction accuracies using ssGBLUP and single-step Bayesian regression methods (SSBR) in 988 genotyped and 1438 non-genotyped Hanwoo cattle, found that the higher accuracies for BFT and MS using ssGBLUP and CWT reached higher accuracies with SSBR. These results suggested that CWT is controlled by a few QTLs with large effects, as shown in Figure 3 and Figure 4. Moreover, Strucken et al. (2017) [22] reported significant QTLs in important regions for this trait in chromosomes 1, 2, 3, 6, 10, and 14.

In this study, WGBLUP outperformed GBLUP for BFT and CWT, where SNPs have large or moderate effects. This is mainly due to the fact that the latter only rely on average relationships among animals across the genome and is thus less sensitive to the genetic architecture of the traits under study [23]. The performance of single-step WGBLUP has been reported to be better than the ssGBLUP and delivered up to 6% points increase in the accuracy of genomic prediction for protein content [11]. Moreover, for traits with identified QTL, weighted ssGBLUP outperformed its unweighted counterpart by 2% to 14% in accuracy of genomic prediction for milk production traits and udder type traits [12] in the Saanen breed. Furthermore, Lourenco et al. (2014) [24] showed that WssGBLUP could outperform GBLUP for traits where QTL have moderate or large effects. On the other hand, traits with polygenic determinism would not benefit from the use of the WGBLUP method, given that only a slight decrease in accuracy was observed when compared to the GBLUP. 

## 4. Conclusions

The present work aimed to improve the accuracy of GBLUP through the use of SNP weighting iterative methods in WGBLUP. Our results indicate that the WGBLUP using a nonlinearA weighting method provides improved but minimal predictions for BFT (4.84%) and CWT (2.70%) in Hanwoo cattle in contrast to the unweighted GBLUP method. Particularly, the WGBLUP using a nonlinearA weighting method with a CT value of 1.25 and exponential limit of 20 at four iterations reached the maximum prediction accuracy for BFT and CWT. In conclusion, the gains from a WGBLUP analysis are still dependent on several factors, such as the method used to obtain the weights, the genetic architecture of the traits (e.g., number of QTL, heritabilities), and the reference population size.

## Figures and Tables

**Table 1:** Heritability and summary statistics for carcass traits of Hanwoo cattle.

Trait	h^2^ (SE)	N	Mean	SD	Min	Max
BFT, mm	0.36 ± 0.02	10,215	14.25	5.03	2.00	47.00
CWT, kg	0.37 ± 0.02	10,215	441.06	52.31	159.00	692.00
EMA, cm^2^	0.35 ± 0.02	10,215	95.61	12.06	34.00	156.00
MS (1–9)	0.45 ± 0.03	10,215	6.10	1.87	1.00	9.00

h^2^, heritability; SE, standard error; SD, standard deviation; CWT, carcass weight; BFT, backfat thickness; EMA, eye muscle area; MS, marbling score.

**Table 2:** Accuracy (Acc) and bias (Reg) of genomic prediction from different methods for backfat thickness (BFT), carcass weight (CWT), eye muscle area (EMA), and marbling score (MS).

Method	BFT		CWT		EMA		MS	
	Acc	Reg	Acc	Reg	Acc	Reg	Acc	Reg
PBLUP	0.37	1.08	0.49	1.19	0.40	1.04	0.37	1.13
GBLUP	0.62	0.97	0.74	1.08	0.67	1.05	0.65	1.18
WGBLUP_linear	0.63	0.72	0.72	0.81	0.64	0.75	0.63	0.86
WGBLUP_nonlinearA	0.65	0.92	0.76	0.99	0.67	1.02	0.65	1.14

## Supplementary Materials

The following are available online at https://www.mdpi.com/2073-4425/10/12/1019/s1. Table S1: Estimates of genetic (above diagonal) and phenotypic (below diagonal) correlations among carcass traits of Hanwoo cattle.
