# Correction: Weighted Genomic Best Linear Unbiased Prediction for Carcass Traits in Hanwoo Cattle. *Genes* 2019, *10*, 1019

**DOI:** 10.3390/genes11091013

**Published:** 2020-08-27

**Authors:** Bryan Irvine Lopez, Seung-Hwan Lee, Jong-Eun Park, Dong-Hyun Shin, Jae-Don Oh, Sara de las Heras-Saldana, Julius van der Werf, Han-Ha Chai, Woncheoul Park, Dajeong Lim

**Affiliations:** 1 Division of Animal Genomics and Bioinformatics, National Institute of Animal Science, Rural Development Administration, Wanju 55365, Korea; irvinelopez@korea.kr (B.I.L.); jepark0105@korea.kr (J.-E.P.); hanha@korea.kr (H.-H.C.); wcpark1982@korea.kr (W.P.); 2 Department of Animal Science and Biotechnology, Chungnam National University, Daejeon 34134, Korea; slee46@cnu.ac.kr; 3 Department of Animal Biotechnology, Chonbuk National University, Jeonju 54896, Korea; sdh1214@gmail.com (D.-H.S.); oh5ow@naver.com (J.-D.O.); 4 School of Environmental and Rural Science, University of New England, Armidale 2351, Australia; sdelash2@une.edu.au (S.d.l.H.-S.); jvanderw@une.edu.au (J.v.d.W.)

The authors wish to make the following corrections to this paper [1]:

In Equations (2) and (3), variable M is used for a matrix of centered genotypes but different variables (Z) are used in equation 4 and in some equations (points 1, 3 and 6) of the iterative steps in the algorithm of the WGBLUP approach for the same matrix. Thus, the authors would like to change the variable Z to M in Equation (4) as well as on the iterative steps in the algorithm of the WGBLUP approach. The correct expression for Equation (4) is given as

$$\hat{u}={DM\prime \;G}^{-1}\hat{g},$$

For the iterative steps in the algorithm of the WGBLUP approach, the correct expressions are given as

1. Set parameters to t = 1, $D_{\left(t\right)}=I$, $G_{\left(t\right)}={\mathrm{MD}}_{\left(t\right)}M\prime \lambda$, where $\lambda =\frac{1}{\sum_{i=1}^{m}2p_{i}\left(1-p_{i}\right)}$;
3. Compute SNP effects as ${\hat{u}}_{\left(t\right)}={\lambda D}_{\left(t\right)}M\prime G_{\left(t\right)}^{-1}\hat{g}$;
6. $G_{\left(t+1\right)}={\mathrm{MD}}_{\left(t+1\right)}M\prime \lambda$ was calculated;

Note that on the above steps, a Z (now M) variable for point 1 and a prime on the Z (now M) for points 3 and 6 were also missing on the original version. Likewise, ${\hat{a}}_{g}\;$was changed to $\hat{g}$ in point 3.

The authors would like to apologize for any inconvenience caused. The changes do not affect the scientific results. The manuscript will be updated, and the original will remain online on the article webpage, with a reference to this correction. 
