# Supplementary material for: Weighted Genomic Best Linear Unbiased Prediction for Carcass Traits in Hanwoo Cattle
Source: Genes (Basel). 2019 Dec 6;10(12):1019. doi: 10.3390/genes10121019 (PMC6947347; doi:10.3390/genes10121019)
Supplement: Supplementary file 1 [file genes-10-01019-s001.pdf]

Supplementary Materials:

Table S1. Estimates of genetic (above diagonal) and phenotypic (below diagonal) correlations among carcass traits of Hanwoo cattle.

Trait	BFT	CWT	EMA	MS
BFT		0.14± 0.10	-0.19± 0.10	0.12± 0.10
CWT	0.37± 0.01		0.50± 0.07	0.30± 0.09
EMA	0.06± 0.01	0.52± 0.01		0.56± 0.07
MS	0.11± 0.01	0.20± .01	0.43± 0.01	
